# Cross-Talk between Iron Deficiency Response and Defense Establishment in Plants

**DOI:** 10.3390/ijms24076236

**Published:** 2023-03-25

**Authors:** Vicente Montejano-Ramírez, Eduardo Valencia-Cantero

**Affiliations:** Instituto de Investigaciones Químico Biológicas, Universidad Michoacana de San Nicolás de Hidalgo, Edifico B3, Ciudad Universitaria, Morelia 58030, Mexico

**Keywords:** iron deficiency in plants, biotrophic pathogens, plant immunity, biotic and abiotic-stress, volatile organic compounds

## Abstract

Plants are at risk of attack by various pathogenic organisms. During pathogenesis, microorganisms produce molecules with conserved structures that are recognized by plants that then initiate a defense response. Plants also experience iron deficiency. To address problems caused by iron deficiency, plants use two strategies focused on iron absorption from the rhizosphere. Strategy I is based on rhizosphere acidification and iron reduction, whereas Strategy II is based on iron chelation. Pathogenic defense and iron uptake are not isolated phenomena: the antimicrobial phenols are produced by the plant during defense, chelate and solubilize iron; therefore, the production and secretion of these molecules also increase in response to iron deficiency. In contrast, phytohormone jasmonic acid and salicylic acid that induce pathogen-resistant genes also modulate the expression of genes related to iron uptake. Iron deficiency also induces the expression of defense-related genes. Therefore, in the present review, we address the cross-talk that exists between the defense mechanisms of both Systemic Resistance and Systemic Acquired Resistance pathways and the response to iron deficiency in plants, with particular emphasis on the regulation genetic expression.

## 1. Introduction

Plants are sessile organisms that are at risk of attack by various pathogens and herbivores. To defend against these attacks, plants have developed two types of defense: Induced Systemic Resistance (ISR) and Systemic Acquired Resistance (SAR) [1]. Induced Systemic Resistance is defined as an increase in defense capacity that is activated in response to necrotrophic pathogens and beneficial microorganisms such as plant growth-promoting rhizobacteria (PGPRs) [2]. Systematic Acquired Resistance is defined as a phenomenon in which unexposed parts of the plants become resistant to infections caused by biotrophic organisms present in any other part of the plant [3].

**Figure 1:**
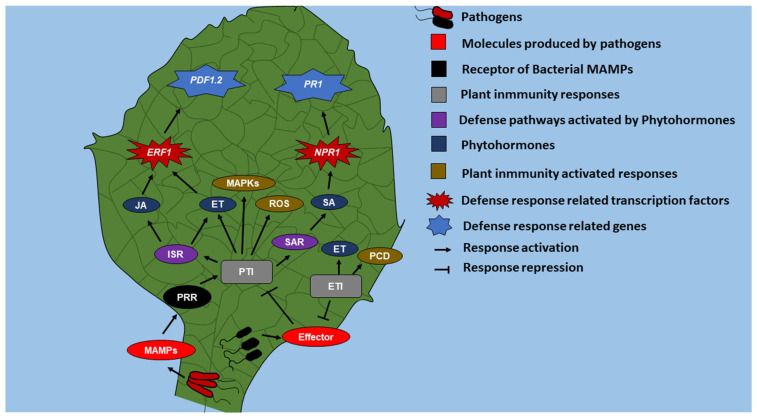
General view of the plant defense mechanism. During pathogenesis, pathogens produce MAMPs which are recognized by plants through PRR to activate PTI and, consequently, induce ET, ROS, and MAPKs production. On the other hand, there are successful pathogens capable of inhibiting PTI through effectors. Plants respond to effectors through the *R* gene to activate ETI, which induces ET production and activation of PCD. During PTI, JA/ET and SA activate ISR and SAR resistance paths, respectively. JA/ET regulates transcription factor *ERF1* expression, which in turn induces *PDF1.2*. Additionally, SA regulates *NPR1* expression, which in turn induces *PR1*. Defense genes, *PDF1.2* and *PR1* are expressed both in the root and plant shoot.

During pathogenesis, pathogens produce molecules with highly conserved structures called Microbe-Associated Molecular Patterns (MAMPs), such as lipopolysaccharides in the outer membrane of gram-negative bacteria, chitin in fungal cell walls [4], flagellin [5] siderophores [6], and antibiotics (2,4-Diacetylphloroglucinol) [7], which are perceived by plants (Figure 1) through Pattern Recognition Receptors (PRR) [8] to activate pattern-triggered immunity (PTI). During PTI, Mitogen-Activated Protein Kinases (MAPKs) is activated, the production of ethylene (ET) increases, and Reactive Oxygen species (ROS) are accumulated [9].

Additionally, some microorganisms are capable of inhibiting PTI through proteins called effectors. Effectors are recognized by the R proteins present in plants, which activate the effector-triggered immunity (ETI) and cause programmed cell death (PCD) or a hypersensitive response and a rapid increase in the production of ET. Effector-triggered immunity is a faster and more robust defense mechanism than PTI [10]. During PTI, there is an increase in the synthesis of phytohormones (in addition to ET) involved in the establishment of ISR and SAR, such as Jasmonic acid (JA) and Salicylic acid (SA), respectively. Jasmonic acid increases the expression of the *Plant Defensin 1.2* (*PDF1.2*) gene through the transcription factor Ethylene Responsive Factor (ERF), activating the ISR defense pathway [11], and the expression of the *Pathogenesis-Related 1* (*PR1*) gene [12] via the transcription factor Non-Pathogenesis-Related 1 (NPR1), which leads to the establishment of SAR [13].

Plants also experience stress caused by abiotic factors such as nutrient availability. Iron (Fe) deficiency is one of the main problems in agriculture because despite being one of the four most abundant elements in the soil, it is found mainly in insoluble forms, such as oxyhydroxide polymers (FeOOH). These Fe (III) oxides are stable, and their solubility is low at neutral pH and in an aerobic environment [14]. In plants, iron is involved in the production of chlorophyll, and hence in the maintenance of chloroplast structure and function, making it essential for photosynthesis [15]. Plants that experience iron deficiency therefore have interveinal chlorosis in young leaves [16].

**Figure 2:**
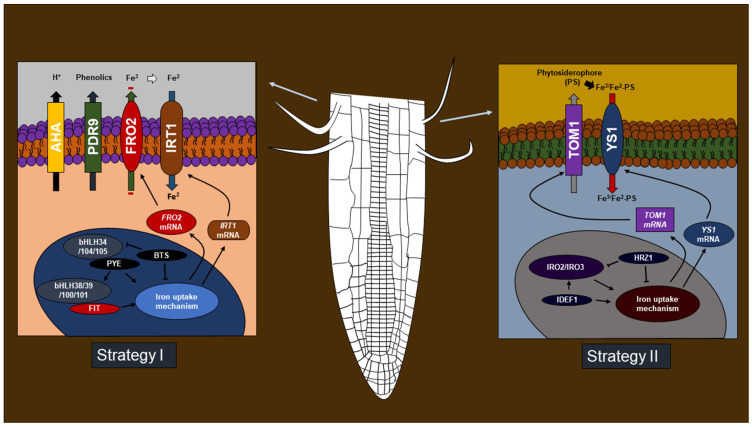
Strategies used by plants for iron uptake. In iron deficiency conditions, Strategy I plants activate proton secretion through AHA for rhizosphere acidification. This mechanism is accompanied by phenol release, which chelates iron. Subsequently, free or chelated iron is reduced by ferric chelate reductase FRO2 and internalized by IRT1. This mechanism is regulated by the transcription factors PYE, bHLH34/38/39/100/101/104/105, and the ubiquitin ligase BTS. In Strategy II plants, phytosiderophores which chelate iron are secreted through TOM1. The chelated iron is internalized by YS1. The Strategy II mechanism is regulated by the transcription factors IRO2/IRO3, IDEF1, and the ubiquitin ligase HRZ1.

To address problems caused by iron deficiency, plants use two strategies focused on iron uptake from the rhizosphere. Strategy I (Figure 2) is used by non-grass monocotyledonous and dicotyledonous plants, to increase iron solubility through rhizosphere acidification by proton release through the enzyme ATPase [17]. Additional elements, such as the secretion of phenolic compounds, carboxylates, and flavonoids from the protein “Pleiotropic Drug Resistance 9” (PDR9) participate during Strategy 1 to chelate Fe (III) [18]. Finally, the free/chelated Fe (III) is reduced to Fe (II) by the action of the enzyme ferric chelate reductase, encoded by the *Ferric Reductase Oxidase 2* (*FRO2*) gene [19], and subsequently internalized into the root cells through the iron-regulated transporter 1 transport protein (IRT1) [20]. The *FRO* and *IRT* genes are regulated by the Fer-Like Iron Deficiency-induced Transcription Factor (FIT) [21]. This FIT is a functional ortholog of FER, a Basic Helix–Loop–Helix (bHLH)-type transcription factor that interacts with additional bHLH transcription factors (bHLH 38/39/100/101) [22]. The expression of the transcription factor bHLH 38/39/100/101 is in turn regulated by bHLH 34/104/105, which forms homodimers or heterodimers [23]. Additionally, bHLH 104/105 interacts with POPEYE (PYE), another bHLH factor involved in the positive regulation of the iron deficiency response. Another element, BRUTUS (BTS), is a putative E3 ubiquitin ligase that negatively regulates the iron-deficiency response [24].

On the other hand, grass monocots use Strategy II for iron uptake (Figure 2), which is based on iron chelation. During this strategy, phytosiderophores (PS) are released, mainly mugineic acid (MA), which chelates Fe (III) [25]. The Fe (III)-PS complex is internalized by the transporter “Yellow Stripe 1” (YS1) [26]. The iron deficiency response in monocotyledons is controlled by the transcription factor IRO2, which regulates the expression of genes involved in PS synthesis and transport [27]. Additionally, the iron deficiency binding factor 1 and 2 transcription factors (IDEF1 and 2) [28] regulate the expression of *IRO2* and several genes involved in iron uptake: *Yellow Stripe Like 15* (*YSL15*), *Yellow Stripe Like 2* (*YSL2*), *Iron Regulated Transporter* (*IRT1*), *Nicotianamine Sintase 2* (*NAS1*), *Nicotianamine Sintase 2* (*NAS2*) and *Nicotianamine Sintase 3* (*NAS3*) [29]. The negative regulation of iron deficiency response in monocotyledons is carried out by a *BTS* homologous gene, called *HRZ1*, which inhibits the expression of *IRO2* and *IRO3* [30]. Phytohormones such as ET [31], SA [32], and JA [33], which activate plant defense responses, also regulate iron uptake response.

At present, there are excellent reviews that explore the interaction between defense and the iron deficiency response in plants. Romera et al., 2019 [34], focus on explaining the molecular overlap that is established between ISR and iron deficiency response, with particular emphasis on the effect of PGPR and plant growth-promoting fungi (PGPF) on the plants. On the other hand, Herlihy et al., 2020 [35], address the competitive interaction for iron availability, established between pathogenic microorganisms and plants, as well as the effect of iron biofortification on plants defense mechanisms, considering that the high availability of this micronutrient in the plant could improve resistance to diseases, through mechanisms such as mitigation of the damage caused by the production of ROS due to the overexpression of ferritin, as well as by the limitation of iron available to pathogens. Liu et al., 2020 [36], review iron homeostasis in the plant and in phytopathogenic bacteria and fungi during the infection interaction, as well as the intra- and intercellular distribution of iron that occurs during the immune response. Therefore, the present work is focused on addressing the cross-talk that exists between the defense mechanisms of both the SAR and ISR pathways and the response to iron deficiency in plants, with particular emphasis on the regulation of the expression of the genes that participate in the previously mentioned pathways, and with the aim to contribute to the comprehension of a phenomenon that constitutes an emerging research area with potential importance in improving cultivation of plants of agricultural interest.

## 2. Molecules Produced during Pathogenic Defense Facilitate Iron Uptake

During pathogenic defense, plants produce various secondary metabolites that are mainly derived from isoprenoids, phenylpropanoids, alkaloids, or fatty acid pathways [37]. Phenolic compounds are the most commonly used antimicrobial agents. Bryophytes produce polyphenols and flavonoids; however, vascular plants produce the largest amounts of these compounds [38]. The accumulation of phenolic compounds in plant tissues is due to an increase in the activity of the enzymes phenylalanine ammonia lyase (PAL) [39] and chalcone synthase (CHS). The activity of phosphoenolpyruvate carboxylase also increases, changing sucrose production to favor defense establishment [40]. Phenolic compounds confer several physiological responses in plants to ensure survival and adaptation to environmental changes [41].

The production of phenolic compounds increases in the presence of pathogens. In pea (*Pisum sativum*) roots infected with the fungus, *Fusarium oxysporum,* an accumulation of phenols was observed in the cell walls and intracellular spaces of the host, as well as on the surface and even within the hyphae of the invading pathogen [42]. However, it was demonstrated that phenols derived from plants inhibited the growth of different species of the genus *Pectobacterium*: *Pectobacterium carotovorum*, *P. brasiliensis*, *P. atrosepticum* and *P. aroidarum* by between 20 and 100% [43].

**Figure 3:**
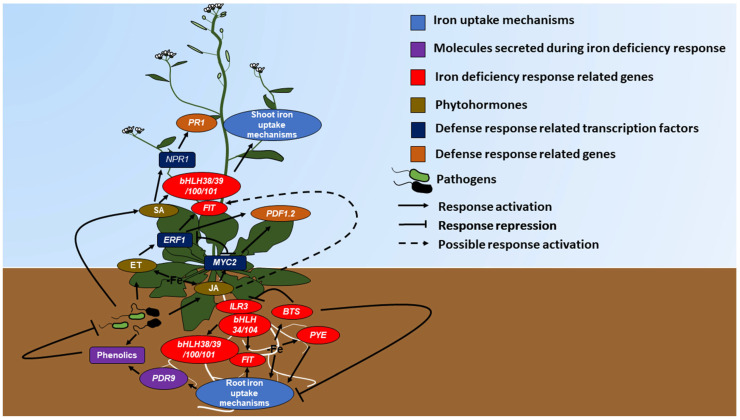
Cross-talk between iron and defense responses. During iron deficiency, plants induce ET, JA, and SA synthesis, and other phytohormones that regulate defense responses in plants. In turn, ET and JA regulates iron uptake by an induction in the expression of *ERF1* and *MYC2* (in the shoot) [53], respectively. These genes encode transcription factors that activate of the defense gene *PDF1.2* (ISR pathway) and the *FIT* gene, a transcriptional regulator of the iron deficiency response pathway (in the shoot). The response is similar when the plant is attacked by a pathogen. During pathogenesis, the plant produces, in addition to phenols, phytohormones such as ET, JA and SA. The SA activate the SAR defense pathway by inducing the expression of the *PR1* gene through *NPR1*, which encode a transcription factor. SA also induces the expression of the *bHLH38* gene that participates in the regulation of the response to iron deficiency pathway. Therefore, when plants perceive iron deficiency, the response machinery to this pathway is activated, through the induction of the expression of genes such as *PYE*, *BTS*, *ILR3*, *bHLH34*/*38/39*/*100*/*101*/*104* and *FIT* [54], in addition to the secretion of phenols through the protein encoded by *PDR9*, which facilitate iron chelation. On the other hand, during iron deficiency response, defense mechanisms are activated to protect plants from pathogens.

In the iron deficiency response, Strategy I (Figure 3) plants release phenolic compounds via the PDR9 protein to chelate iron [18]. Iron deficiency in *Arabidopsis thaliana* increases the expression of genes involved in the synthesis and secretion of phenolic compounds, in addition to the upregulation of the *PDR9* gene. In *pdr9* mutants, plant growth decreases under iron deficiency and downregulates the expression of *IRT1* and *FRO2* [44]. In the case of Strategy II plants, the phenolic efflux zero protein (PEZ1), located in the plasma membrane and involved in the efflux of protocatecholic acids (PCA) and caffeic iron chelators, is also induced by the absence of iron. Transgenic plants overexpressing PEZ1 grow better in soils with high pH and low iron availability [45]. Additionally, the antimicrobial effects of protocatecholic acid have been evaluated. The growth of *Bacillus thuringiensis kurstaki* is lower in iron-deficient conditions, and during this stationary phase, the *asbF* gene, involved in the synthesis of PCA, is expressed, indicating that this compound sequesters iron and inhibits microbial growth [46]. In contrast, the PCA from *Veronica montana* L. has an antimicrobial effect against *Staphylococcus aureus*, *Bacillus cereus*, *Pseudomonas aeruginosa*, *Microccocus flavus*, *Listeria monocytogenes*, *Enterobacter cloacae* and *Escherichia coli* [47]. The isolated PCA of *Paenibacillus elgii* HOA73 has a potent antifungal effect against *Botrytis cinerea* and *Rhizoctonia solani* [48], and finally, caffeic acid inhibits bacteria such as *Bacillus subtilis*, *Escherichia coli*, *Pseudomonas fluorescens*, *Staphylococcus aureus*, fungi such as *Aspergillus niger*, *Candida albicans* and *Trichophyton rubrum* [49] and viruses such as hepatitis C [50] and influenza A [51]. More information on antimicrobial phenols and particularly coumarins production during iron deficiency can be found in the literature review of Stringlis et al. (2019) [52].

These data demonstrate that during the establishment of the defense response, the plant simultaneously activates iron uptake pathways. The plant produces various molecules with inhibitory activity against fungi, bacteria, and viruses, which, in turn, chelate and solubilize iron. These regulatory effects on defense responses and iron deficiency occur through an increase in the expression of genes such as *IRT1*, *FRO2* and *PDR9* that encode key proteins for iron uptake. Due to the above, the cross-talk of both pathways at the genetic level is highlighted.

## 3. Phytohormones Favor Iron Deficiency Response

The functions of phytohormones in the development and defense of plants are well known. Auxins [55], gibberellic acid (GA) [56], brassinosteroids [57], and cytokinins (CKs) are among the phytohormones related to development and plant growth [58]. Although their main effect is on growth, these phytohormones also modulate defense responses in plants [59,60,61,62]. Phytohormones mainly involved in systemic resistance pathways are JA, ET [63], SA [64], and abscisic acid (ABA) [65].

In addition to participating in development and defense, phytohormones facilitate iron uptake (Figure 3). Iron deficiency increases auxin synthesis in *Arabidopsis*, which in turn increases *FIT* and *FRO2* (Table 1) expression. Additionally, the exogenous application of auxins stimulates the transcription of these genes [66]. Moreover, ethylene production in cucumber (*Cucumis sativus* L.), tomato (*Lycopersicon esculentum* Mill.), and peas (*Pisum sativum* L.) grown under iron deficiency were higher [67]. Treatment of *Arabidosis* and tomato with 1-aminocyclopropane-1-carboxylate (ACC; a precursor of ethylene) increased *FIT*, *FRO,* and *IRT* expression. The application of ethylene inhibitors to plants grown under iron-limiting conditions represses the expression of these genes [31]. Subsequently, it was demonstrated that iron deficiency increases *AtSAM1*, *AtSAM2*, *AtACS4*, *AtACS6*, *AtACS9*, *AtACO1*, and *AtACO2* expression, which are involved in ethylene synthesis, and *AtETR1*, *AtCTR1*, *AtEIN2*, *AtEIN3*, *AtEIL1,* and *AtEIL3*, which are involved in ET signaling [68]. Regarding the function of JA in iron deficiency response establishment, Maurer et al. (2011) [69] proposed this phytohormone as a negative regulator because *Arabidopsis* plants grown under iron-deficient conditions and treated with 100 μM of methyl jasmonate presented lower expression of *AtFRO2*, *AtFIT* and *AtIRT1*. In another study, Montejano-Ramírez et al. (2020) [70], also showed that treatment of *Medicago truncatula* with 20 μM JA lowered the expression of the iron deficiency response genes *MtbHLH38*, *MtbHLH39*, *MtFIT*, and *MtFRO3*, which was reflected in a decrease in the chlorophyll content. The treatment of *A. thaliana* plants with SA increased *AtbHLH38* and *AtbHLH39* expression, which are key to the establishment of the iron deficiency response [32]. However, *M. truncatula* treated with 100 μM SA showed reduced gene expression in response to iron deficiency [70]. The addition of SA also increased the chlorophyll content in peanut plants (*Arachis hypogaea*) grown under conditions of iron sufficiency and deficiency [71], indicating an opposing result in comparison to that of *M. truncatula* [70].

Similarly, in Strategy II plants, ET production in rice roots grown under iron-deficient conditions increased, and ACC treatment conferred tolerance to this metal deficiency. It was also demonstrated that *OsIRO2*, *OsNAS1*, *OsNAS2*, *OsYSL15,* and *OsIRT1* expression increased, indicating that ET is involved in the positive regulation of iron uptake mechanisms [76]. Kobayashi et al. (2016) [33], using rice plants grown under iron-deficient conditions, showed that several genes induced by JA are also negatively regulated by the ubiquitin ligases “Hemerythrin motif-containing really interesting new gene (RING)-and zinc-finger protein 1/2”(OsHRZ1/2) and positively regulated by IDEF1 transcription factors. Additionally, an increase in JA content in transgenic plants silenced in *OsHRZ1* (*iHRZ1*) expression grown under iron-sufficient conditions was observed. In non-transgenic plants, iron deficiency per se increased JA and jasmonoyl isoleucine concentrations. Therefore, under these conditions, JA induces *IDEF1* expression, which in turn increases JA synthesis, and thus increases the expression of genes involved in iron uptake and translocation. These results are contradictory to those shown by Maurer et al. (2011) [69], in which JA was proposed as a negative regulator of iron uptake.

Based on the above, it is proposed that iron deficiency in plants increases the synthesis of phytohormones as an accessory mechanism to increase the expression of genes involved in the codification of iron reduction and uptake protein. Therefore, the effect of defense phytohormones on the response pathway to iron deficiency occurs at the genetic level.

## 4. Iron Deficiency Induces Defense Gene Expression

In addition to antimicrobial compound production during the iron deficiency response [18] that facilitates iron uptake and defense establishment by plants, it has been shown that in plants grown under iron-deficient conditions, the expression of genes related to defense increases (Figure 3). Wheat plants grown in the presence of Fe (III) and infected with *Blumeria graminis* f. sp. Tritici (Bgt) showed an increase in *PR1a* and *PR1b* expression, since pathogen presence causes exhaustion of plant intracellular iron, which in turn causes iron deficiency. The results were similar when adding deferoxamine, an iron chelator [77]. Subsequently, treatment of *Arabidopsis* plants with the bacterial siderophore crisobactin induced *AtPR1* and *AtPAD4* expression. This is due to the iron deficiency caused by siderophores in the plant [73]. Additionally, in this plant, it was demonstrated that the inoculation of *Dickeya dadantii* in conjunction with iron deficiency increased *AtPR1* expression and decreased the expression of pectato lyase genes (*PelA*, *PelB*, *PelC,* and *PelD*) that are involved in the development of infection symptoms. Iron deficiency also reduced infection symptoms caused by the *fungus Botrytis cinerea* [74] and induced the expression of the *AtPR1* and *AtPDF1.2* genes [72]. Iron deficiency leads to the induction of *MPK3*/*MPK6* expression, whose proteins participate in ACS2/ACS6 phosphorylation, which are enzymes involved in the ET synthesis pathway, a key phytohormone for ISR establishment [78]. Finally, it was also shown that the iron deficiency in *M. truncatula* induced the expression of defense genes *MtDef2.1* and *MtPR1* [75].

In Strategy II plants, it has been observed that the expression of the *OSRMC* gene induced by JA increases in iron deficiency [79] and that 10 of 35 genes involved in phytohormone synthesis are also induced under iron-limiting conditions [33].

Iron deficiency regulates the expression of several genes involved in plant defense pathways, indicating that during abiotic stress such as iron deficiency, the plant also establishes a defense mechanism to face the possible vulnerability to which it is exposed by the lack of nutrients. It should be noted that all this regulation of pathways occurs through interaction between defense response and iron deficiency responsive genes, specifically those that encode transcription factors.

## 5. Microorganisms Activate Iron Deficiency Response and Defense Pathways

The plant root system, whose function is anchorage, as well as the intake of nutrients and water, also produces compounds that stimulate growth and regulate interactions with soil microorganisms, creating a denominated “rhizosphere” zone around the roots [80,81]. In the rhizosphere, PGPR and PGPF promote plant growth through mechanisms that include the mineralization and transformation of nutrients [82,83,84]. Some PGPR improve iron uptake by increasing the secretion of molecules with chelating capacity [85,86]. The PGPR and PGPF also protect plants against microbial attacks, either by antagonizing them [87] or by activating defense pathways (ISR or SAR) [1,82,88].

Different studies have demonstrated the ability of PGPR to activate both iron deficiency and biotic stress response pathways. *A. thaliana* plants inoculated with the bacterium *Paenibacillus polymyxa* BFKC01 showed an increase in the expression of the *FIT1*, *FRO2*, and *IRT1* genes, while an induction was also observed for the *PR1*, *PR2* and *PDF1.2* genes [89]. Another bacterium with this capacity is the *Arthrobacter* sp. UMCV2, which, in addition to promoting the growth of *M. truncatula*, also increased the expression of *MtFRO2*, *MtFRO3*, *MtFRO4*, *MtFRO5*, *MtDef2.1* and *MtPR1* genes [75]. Additionally, inoculation of *Serratia marcescens* NBRI1231, a PGPR bacterium, in bethel plants (*Piper betle*) infected with *Phytophthora nicotianae*, increased phenol content, mainly gallic, protocatechuic, chlorogenic, caffeic, ferulic, and ellagic acids [90].

Plant growth-promoting fungi also solubilize phosphates and produce Indole acetic acid (IAA), cellulose, chitinase, and siderophores, which chelate iron and allow its uptake by plants [91,92,93]. Hossain et al. (2017) [94] determined that *Penicillium viridicatum* GP15-1 triggers ISR in *A. thaliana*, restricting the growth of *Pseudomonas syringae* pv. *Tomato* DC300 and development of the disease. In another study, Murali et al. (2013) [95] observed that susceptible pearl millet seeds (cultivar 7042S) treated with spores of *Penicillium chrysogenum* (PenC-JSB9) induced resistance to downy mildew caused by *Sclerospora graminicola*. PenC-JSB9 treatment reduced disease incidence (by 28%) compared to untreated controls. In Northern blot analysis, PenC-JSB9 pretreated susceptible seedlings showed rapid and enhanced expression of the defense-related genes *LOX*, *POX*, and *CHT*. Enhanced activation of defense genes by PenC-JSB9 suggests a role in elevated resistance against *S.graminicola*.

Treatment with both PGPR and PGPF regulates defense responses in plants while improving growth by modulating nutrient uptake, either by inducing gene expression or by the production of secondary metabolites, such as siderophores. The antecedents indicate a joint regulation of the response to biotic and abiotic stress mediated by PGPR and PGPF, indicating feedback between these pathways.

## 6. Volatile Organic Compounds Regulates Defense and Iron Deficiency Response

Both PGPR and PGPF also regulate plant growth through the emission of Volatile Organic Compounds (VOCs) [96,97,98]. Hence, some microorganisms may activate iron deficiency responses and defense pathways by VOCs, such as the fungi *Trichoderma asperellum* and *T. harzianum*, whose VOCs increased the expression of the *AtbHLH38*, *AtbHLH39* genes, *FRO2* and *IRT1*, which are involved in the uptake of iron and the *PDF1.2* gene of the ISR pathway, thus improving the resistance of *A. thaliana* to the necrotrophic fungus *B. cinerea*. This effect was similar in *Solanum lycopersicum* [99].

Subsequently, induction of the *MYB72* gene was observed both in conditions of iron deficiency and in the presence of volatile organic compounds produced by the bacterium *Pseudomonas simiae* WCS417. The transcription factor MYB72 regulates both the establishment of ISR and the synthesis and secretion of phenolic compounds to facilitate iron chelation and mobilization during iron deficiency response [100]. Among phenolic compounds, scopoletin modifies the ensemble of the rhizospheric microbial community, selectively inhibiting soil-borne fungal pathogens such as *F. oxysporum* and *Verticillium dahliae*, but not the PGPR *Pseudomonas* WCS417 [101].

Concordantly, Hernández-Calderón et al. [102] demonstrated that VOCs produced by bacteria with different lifestyles, including *Arthrobacter* sp. UMCV2, *Bacillus methylotrophicus* M4-96, *Sinorhizobium meliloti* 1021, the plant pathogen *Pseudomonas aeruginosa* PAO1, and the commensal rhizobacterium *Bacillus* sp. L2-64, increased biomass and chlorophyll content and improved the root architecture of *Sorghum bicolor*, except for commensal bacteria. Additionally, the expression of iron uptake genes *SbIRT1*, *SbIRT2*, *SbYS1*, and *SbYS2* was evaluated. The expression of the *SbIRT1* gene increased in response to volatiles of *Arthrobacter* sp. UMCV2 (35-fold), *P. aeruginosa* PAO1 (56-fold), *Bacillus sp*. L2-64 (35-fold), and *S. meliloti* 1021 (140-fold). However, the expression of the *SbYS1* and *SbIRT2* genes was regulated by the VOCs of *P. aeruginosa* PAO1, *Bacillus* sp. L-254, and *S. meliloti* 1021, but no effect was observed with *Arthrobacter* sp. UMCV2. In contrast, the VOCs of *B. methylotrophicus* M4-96 decreased the expression of all genes evaluated. In the case of the defense genes, *PR1* (from the SAR pathway) and COI1 (from the ISR pathway), only PGPRs *A. agilis* UMCV2 and *S. meliloti* 1021 as well as the pathogenic *P. aeruginosa* PAO1 increased their expression. On the other hand, the expression of COI1 was only increased by the VOCs of the PGPRs. This indicates that plants recognize bacteria through VOCs and by activating defense pathways. However, they also demonstrate activation of the iron uptake pathway, even when plants are treated with VOCs from phytopathogenic bacteria, which highlights the cross-linking between the iron deficiency response and defense pathway.

*Arthrobacter* sp. UMCV2 and *S. meliloti* 1021 emit *N*,*N*-dimethylhexadecylamine (DMHDA) [96,103], a VOC that promotes the growth of plants such as *S. bicolor*, *A. thaliana,* and *M. truncatula* [97,98,104]. Plants of *M. truncatula* exposed to DMHDA trigger iron deficiency responses, including rhizosphere acidification and ferric iron reduction, even when plants are grown in iron-sufficient conditions [97]. DMHDA also induces JA signaling in Arabidopsis, as has been shown with the induction of the JA-responsive gene markers *pLOX2:uidA* and *JAZ1/TIFY10A-GFP* [105,106]. In an integrative study [70], plants of *M. truncatula* exposed to DMHDA experienced a growth 1.5-fold higher than that of unexposed plants under conditions of iron sufficiency or deficiency. Under iron sufficiency conditions, DMHDA induced the expression of iron deficiency responsive genes *MtbHLH38*, *MtbHLH39*, *MtFIT,* and *MtFRO3* 2.4- to 4.4-fold higher than that of the controls. Nevertheless, plants treated with DMHDA combined with iron deprivation showed 4.7- to 52.2-fold higher expression than that of the controls. In plants exposed to DMHDA in iron sufficiency conditions, SAR-related genes, *MtNPR4* and *MtWRKY70,* and *IRS*-related genes, *MtMYC2* and *MtDef2.1,* showed higher expression compared with the controls, and the effect was synergistic when DMHDA was combined with iron deficiency. However, iron deficiency turns on genes related to the defense of the SAR and ISR pathways, whereas the addition of JA and SA does not turn on but rather represses genes related to responses to iron deprivation. This study clearly showed the asymmetrical cross-talk of iron and defense responses triggered by a fully identified bacterial VOC as DMHDA.

## 7. Discussion

Several studies have shown that antimicrobial molecules participate during iron deficiency response establishment [18,44,45]; therefore, during the stress process due to iron deficiency, the plant defends itself against attack of various microorganisms [72,74], establishing a competitive process for iron uptake due to the importance of this metal for vital metabolic processes [15]. Antimicrobial compounds produced during this type of abiotic stress are generally phenolic in nature. Phenolic compounds kill fungal pathogens by altering cell membrane permeability, disrupting cell wall integrity, suppressing enzyme activity, free radical formation, inhibiting biosynthesis of certain proteins, damaging DNA, and suppressing expression of virulence genes. In the case of viral infection in plants, phenols repress virus replication through protein, DNA, or ribose nucleic acid (RNA) damage, inhibiting viral enzyme activities, viral RNA translation and viral DNA replication as well as protein synthesis and transcription factors responsible for viral enzymes [107].

On the other hand, the attacking microorganism produces siderophores that sequester iron and make its uptake difficult for the plant, during which the plant activates an iron deficiency mechanism and, in turn, defends itself from the pathogen through induction of defense gene expression [73]. Siderophores act on the plant’s immune response in two different ways: decreasing the iron content, which results in direct activation of the defense, or as a priming mechanism for defense responses. Several lines of evidence indicate that siderophores such as EDDHA activate the salicylic acid pathway in *A. thaliana* through iron deficiency in plant. A transcriptomic analysis of siderophore-treated leaves indicated that the most overrepresented function in differentially expressed genes was immunity. Therefore, treatment of plants with siderophores clearly mimics biotic stress [52].

Unlike pathogens, whose induction of iron deficiency response gene expression is caused by competition with the plant for the availability of this metal, plant growth-promoting microorganisms such as PGPR and PGPF increase the gene expression mainly through the emission of VOCs, without presenting any negative effect on the plant which is reflected in growth promotion and a higher chlorophyll content in the plant [96,97,98]. Additionally, these microorganisms protect the plant from pathogen attack by activating defense pathways through the induction of *PDF* and *PR* genes [70,99,100,102]. On the other hand, when plants are inoculated in the root with these microorganisms, the induction effect on gene expression of both defense pathways and responses to iron deficiency is preserved [75,89,90], which favors the use of these organisms in sustainable agricultural practices.

Both the presence of pathogens and promoter microorganisms activate the synthesis of phytohormones in the plant; therefore, a hypothesis to explain the regulation between the response to iron deficiency and the establishment of defense in plants involves phytohormones, which regulate various plant processes, including ISR and SAR establishment, and induce mechanisms related to iron uptake [31,32,33]. Iron deficiency increases the production of auxins, ET, and nitric oxide (NO) in plant roots, so it was believed that iron deficiency response was regulated by the individual action of these phytohormones; however, it is known that some phytohormones affect the synthesis of others. ET and auxins stimulate nitric oxide accumulation in the roots of iron-deficient plants, resulting in a stabilization of the FIT transcription factor [108]. It is well known that ET positively regulates the response to iron deficiency in Strategy I plants and in rice, a plant with elements of both Strategy I and II [109]. The role of other phytohormones such as JA and SA in the response to iron deficiency are not clear. JA regulates the response to iron deficiency in plants Strategy I; however, in rice, it activates the expression of some genes only in very early stages of iron deficiency. On the other hand, iron deficiency also increases the synthesis JA, the main phytohormone of ISR pathways [33]. The role of SA in the response to iron deficiency is ambiguous. In lines of *A. thaliana* overexpressing the transcription factors OBF-BINDING PROTEIN 3 (OBP3) inducible by SA, an induction in the expression of the *bHLH38* and *bHLH39* genes is observed [32]. Additionally, the application of exogenous SA in *A. thaliana* induces the expression of the *YSL1* and *YSL3* genes involved in iron translocation and homeostasis [110] On the other hand, iron deficiency also increases the SA content in shoots and roots of *A. thaliana* [111] Another background highlights that SA signaling through NPR1 does not affect the response to iron deficiency [112].

Additionally, the accumulation of NO increases after the attack of pathogens and occurs rapidly during the hypersensitive response [113]. NO modulates the SAR defense pathway by regulating SA-linked proteins such as non-expressor of pathogenesis-related genes (*NPR-1* and *NPR-2*) and group D bZIP (basic leucin zipper domain transcription factor) [114]. NO also contributes positively to the production of JA through an increase in the expression of *LOX3*, *OPR1*, *OPR2,* and *OPR3* genes involved in the biosynthesis of this hormone [115].

With the previously mentioned background, the joint activation of the response to iron deficiency and of the defense pathways regulated by phytohormones can be considered to be caused by the action of NO. In the case of JA and SA, more studies are required to clarify the role of these phytohormones in regulating the response to iron deficiency.

Another hypothesis for the joint regulation of genes for defense and iron deficiency responses is through the mediator complex. Yang et al. (2014) [116], showed that the MED16 subunit interacts with the MED25 subunit, which regulates iron homeostasis by interacting with EIN3 and EIL1, two transcription factors in ethylene signaling associated with the regulation of iron response. Therefore, the mutants in *MED16* and *MED25* showed decreased expression of *FIT*, *IRT,* and *FRO2* genes. Additionally, MED25 interacts with the transcription MYC2 through the TAD domain [117]. Therefore, the defense and iron deficiency response pathways are related through MED16, because this MED subunit is essential in the SA and JA signaling pathways [118] although additional research is needed to elucidate the extent of this relationship. Given all the evidence previously shown in this review, defense and iron deficiency responses should be considered as phenomena that occur together and that depend on each other for regulation through genetic elements present in both pathways. This can help to establish strategies for resistance to biotic and abiotic stresses in the cultivation and conservation of plants of agricultural interest.

## Figures and Tables

**Table 1:** List of genes involved in the cross-talk between defense and iron deficiency responses.

Gene	Pathway	Regulated by	Reference
*ERF1*	ISR	Up-regulated by ET and JA. Down-regulated by MYC2 transcription factor	[11]
*MYC2*	ISR	Up-regulated by JA and iron deficiency	[53,70]
*PDF1.2*	ISR	Up-regulated by ET and JA through ERF1 and MYC2 transcription factors. Up-regulated iron deficiency	[11,72]
*NPR1*	SAR	Up-regulated by SA	[13]
*PR1*	SAR	Up-regulated by SA through NPR1 transcription factor. Up-regulated by iron deficiency	[12,73,74,75]
*PDR9*	* IDR and ** DR	Up-regulated by iron deficiency	[18]
*FIT*	IDR	Up-regulated by iron deficiency. Up-regulated by ET through ERF transcription factor. Down-regulated by JA. Possible up-regulation by JA in early iron deficiency stages in Strategy II plants	[21,33,68,69,70]
*ILR3*	IDR	Up-regulated by iron deficiency. Down-regulated by BTS	[54]
*bHLH34*	IDR	Up-regulated by iron deficiency	[23]
*bHLH104*	IDR	Up-regulated by iron deficiency	[23]
*bHLH38*	IDR	Up-regulated by iron deficiency. Up-regulated by SA. Down-regulated by JA and SA	[22,23,32,70]
*bHLH39*	IDR	Up-regulated by iron deficiency. Up-regulated by SA. Down-regulated by JA and SA	[22,23,32,70]
*bHLH100*	IDR	Up-regulated by iron deficiency	[22,23]
*bHLH101*	IDR	Up-regulated by iron deficiency	[22,23]
*BTS*	IDR	Up-regulated by iron deficiency	[24]
*PYE*	IDR	Up-regulated by iron deficiency	[24]

* IDR: Iron deficiency response; ** DR: Defense response.

## Data Availability

No new data were created or analyzed in this study. Data sharing is not applicable to this article.
